# Hybrid tungsten oxyselenide/graphene electrodes for near-lossless 2D semiconductor phase modulators

**DOI:** 10.1038/s41377-025-02058-8

**Published:** 2026-01-03

**Authors:** Shi Guo, Sung-Gyu Lee, Xiangxin Gong, Lalit Singh, Rui Yu, Ahmad Sholehin Bin Juperi, Seoungbum Lim, Yuhui Yang, Jinpeng Huo, Jeremy Leong, Ce Liang, Hyojin Seung, Yangchen He, Daniel Rhodes, Min Sup Choi, Takashi Taniguchi, Kenji Watanabe, Wonkeun Chang, Beng Kang Tay, Luigi Ranno, Juejun Hu, Qingyun Wu, Lay Kee Ang, Jia Xu Brian Sia, Sang Hoon Chae

**Affiliations:** 1 School of Electrical and Electronic Engineering, Nanyang Technological University, Singapore, Singapore; 2 CNRS-International-NTU-Thales Research Alliance (CINTRA), Singapore, Singapore; 3 Pritzker School of Molecular Engineering, University of Chicago, Chicago, IL USA; 4 Department of Materials Science and Engineering, University of Wisconsin, Madison, WI USA; 5 Department of Physics, University of Wisconsin, Madison, WI USA; 6 Department of Materials Science and Engineering, Chungnam National University, Daejeon, Republic of Korea; 7 Research Center for Materials Nanoarchitectonics, National Institute for Materials Science, Tsukuba, Japan; 8 Research Center for Electronic and Optical Materials, National Institute for Materials Science, Tsukuba, Japan; 9 Department of Materials Science and Engineering, Massachusetts Institute of Technology, Cambridge, MA USA; 10 Science, Mathematics and technology, Singapore University of technology and design, Singapore, Singapore; 11 School of Materials Science and Engineering, Nanyang Technological University, Singapore, Singapore

**Keywords:** Integrated optics, Silicon photonics

## Abstract

Optical phase modulators are critical components in integrated photonics, but conventional designs suffer from a trade-off between modulation efficiency and optical loss. Two-dimensional materials like graphene offer strong electro-optic effects, yet their high optical absorption at telecom wavelengths leads to significant insertion losses. Although monolayer transition metal dichalcogenides (TMDs) provide exceptional telecom-band transparency for low-loss electro-refractive response, their practical implementation in phase modulators requires top electrodes to enable vertical electric field tuning, which typically introduces parasitic absorption. Here, we address this challenge by developing hybrid tungsten oxyselenide/graphene (TOS/Gr) electrodes that minimize optical loss while enabling efficient phase modulation in TMD-based devices. The UV-ozone-converted TOS (from WSe_2_) acts as a heavy p-type dopant for graphene, making the graphene transparent in the NIR region while enhancing its conductivity. Our complete device integrates a hybrid TOS/graphene transparent electrode with a hexagonal boron nitride dielectric spacer and monolayer WS_2_ electro-optic material on a SiN microring platform. This achieves a high modulation efficiency of 0.202 V·cm while maintaining an exceptionally low extinction ratio change of just 0.08 dB, demonstrating superior performance compared to modulators employing conventional electrodes. Our breakthrough in near-lossless phase modulation opens new possibilities for energy-efficient optical communications, photonic computing, and fault-tolerant quantum networks.

## Introduction

Silicon photonics provides a scalable and efficient platform for integrating optical and electronic functionalities onto a single chip. This addresses the rapidly growing demand for high-speed data communication, advanced information processing, and compact optical systems^1,2^. As core components of silicon photonics, electro-optic modulators enable dynamic modulation of optical signals through external electric fields^3^. Traditionally, materials such as doped silicon, germanium, and III–V semiconductors have been employed for this purpose^3–6^. However, these conventional materials inherently suffer from significant drawbacks, including high optical losses, substantial power consumption, large device footprints, and limited modulation efficiencies. These limitations severely restrict their scalability and practicality for high-performance, energy-efficient integrated photonic circuits. Consequently, alternative electro-optic materials are being explored to deliver efficient, low-loss, and compact phase modulation^7–9^.

Recently, two-dimensional (2D) materials have emerged as promising candidates for integration into silicon photonic systems, primarily owing to their extraordinary electronic and optical properties, as well as their atomic-scale thickness^10–13^. Among various 2D materials, monolayer transition metal dichalcogenides (TMDs) are distinguished by their nearly transparent nature and negligible optical absorption in the telecommunication band^14,15^, making them well-suited for low-loss optical phase modulators. In practical use, electro-optic modulation requires additional transparent electrode materials, including carbon nanotube networks^16^, metal nanogrids^17^, and indium tin oxide (ITO)^18,19^. Compared with these bulky electrodes, graphene is superior in terms of uniform conductivity, low contact resistance, high carrier mobility for high-speed modulation, and its atomic-scale compatibility with other 2D materials, which enable stronger electro-optic coupling and more efficient refractive index control^20–23^. However, graphene’s low-energy states near the Fermi level result in considerable optical absorption at telecom wavelengths. This absorption causes insertion losses and compromises modulation efficiency and overall device performance^24–27^. Although ionic liquid gating is capable of injecting significantly high charge densities without introducing absorption losses, it suffers from nonlocal charge dispersion, limited chemical stability, and incompatibility with scalable CMOS fabrication processes^28–30^. Therefore, developing electrode materials that simultaneously achieve efficient electrostatic control, optical transparency, and scalability remains a critical challenge for integrated photonic applications.

In this study, we demonstrate optical loss elimination in photonic electrodes through an innovative graphene doping approach using tungsten oxyselenide (TOS), achieved by oxidizing monolayer WSe_2_. Our hybrid design enables a near-lossless electrode platform with heavily p-doped graphene due to TOS^31–33^, with minimal absorption characteristics at telecommunication wavelengths around 1550 nm. The complete phase modulator integrates a hybrid TOS/graphene transparent electrode with a hexagonal boron nitride (hBN) dielectric spacer and monolayer WS_2_ electro-optic material on a silicon nitride (SiN) microring platform. The optimized heterostructure (HS) maintains telecom-band transparency while delivering robust electro-optic modulation, overcoming the critical loss-modulation compromise in photonic devices. It provides a promising route for advanced photonic integrated circuits with improved modulation efficiency and reduced power consumption.

## Results

**Fig. 1:**
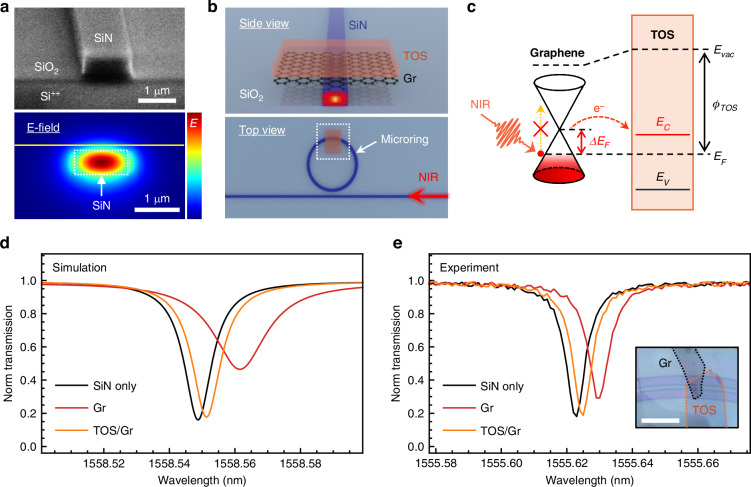
**Substantially reduced absorption of TOS/graphene on waveguides**. **a** SEM image (top panel) of the fabricated SiN waveguide on a SiO_2_/Si substrate, and the simulated electric field distribution (bottom panel) within the waveguide, showing the confined transverse electric (TE) mode. **b** Schematic of the microring resonator integrated with the TOS/graphene HS. **c** Energy band diagram of the TOS/graphene interface, highlighting the electron transfer from graphene to TOS. **d** Simulated transmission spectra of the microring resonator for three different configurations: bare SiN microring resonator, microring integrated with graphene, and microring integrated with the TOS/graphene HS. **e** Experimental transmission spectra validating the simulation results. Shown are the transmission spectra of a bare microring, a microring integrated with graphene, and a microring integrated with TOS/graphene. Inset: Optical image of the TOS/graphene layer on top of the microring resonator; scale bar: 10 µm

We employed SiN as the material for waveguides and microring resonators. Compared with conventional silicon waveguides, SiN waveguides exhibit superior performance due to significantly lower optical losses, broader transparency extending from visible to mid-infrared wavelengths, and excellent compatibility with standard CMOS fabrication processes^34,35^. Figure 1a presents a scanning electron microscopy (SEM) image of the SiN waveguide (top) alongside its simulated electric field distribution (bottom). The transverse electric (TE) mode is predominantly guided within the waveguide, exhibiting an exponential decay outside the waveguide boundaries. This evanescent field enables strong interaction with the materials deposited on top of the waveguide, allowing modulation of the guided mode via modulation of the refractive index of the deposited materials.

To investigate the near-infrared (NIR) absorption properties of the TOS/graphene HS, we employed the microring resonator, which is designed to be highly sensitive to environmental refractive index changes. TOS/graphene HS was transferred onto the microring by the all-dry transfer method (Fig. 1b). Guided light resonating within the microring interacts with the HS layer on top. TOS (5.6 eV) has a significantly higher work function compared to graphene (4.6 eV)^31,32,36^, which drives electron migration from graphene into TOS. This charge transfer results in substantial p-doping of the graphene (Fig. 1c and Supplementary Information III). Due to p-doping, graphene’s Fermi level shifts further into the valence band, increasing the energy required for electrons to transition into the conduction band. As a result, incident NIR photons at telecommunication wavelengths cannot meet these energy-momentum matching conditions, significantly eliminating interband optical absorption^20^.

To investigates the optical behavior of microring resonators, finite-difference time-domain (FDTD) simulations were carried out under three distinct configurations: a bare microring, a microring integrated with pristine graphene, and a microring integrated with a TOS/graphene HS (Fig. 1d). The simulation results reveal that the introduction of pristine graphene leads to both a measurable phase shift and significant optical insertion loss, attributed to its inherent absorption at NIR wavelengths. In contrast, in the heavily p-doped graphene model (TOS/graphene), the microring exhibits a clear phase shift with negligible insertion loss. To verify these predictions, we transferred pristine graphene and TOS/graphene HS (Fig. 1e inset) onto separate microring resonators and measured their transmission spectra (Fig. 1e). In agreement with our calculations, the pristine graphene induces a pronounced reduction in transmission, consistent with increased absorption. In contrast, the TOS/graphene-integrated microring only exhibits a comparable phase shift without any noticeable degradation in transmission intensity. These results clearly confirm that the TOS layer enables optical transparency of graphene at telecom wavelengths, dramatically reducing near-infrared absorption and facilitating near-lossless phase modulation.

**Fig. 2:**
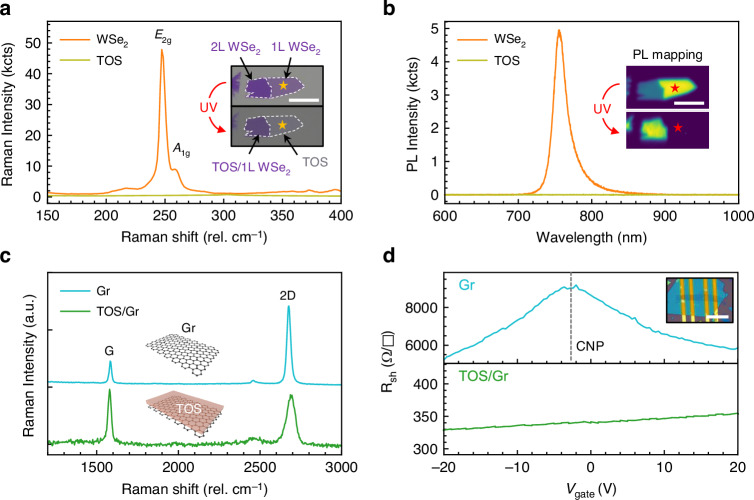
**Optical characterization of TOS and TOS/graphene**. **a** Raman spectra of WSe_2_ flakes before and after complete oxidation, illustrating the disappearance of characteristic WSe_2_ peaks (*E*_2g_ and *A*₁_g_) post-oxidation. Inset: Optical image showing the WSe_2_ flake composed of monolayer and bilayer regions. **b** Photoluminescence (PL) spectra of the same WSe_2_ flake before and after oxidation. Inset: Spatially resolved PL mapping, indicating complete oxidation of monolayer WSe_2_ and partial oxidation of the bilayer. **c** Comparison of Raman spectra for pristine graphene and the TOS/graphene HS. A significant decrease in the intensity ratio of 2D to G peaks is observed, indicating heavy p-doping of graphene by the TOS layer. **d** Sheet resistance (*R*_sh_) measurements of pristine graphene and TOS/graphene HS as a function of gate voltage (*V*_gate_). Upon doping, the charge neutrality point (CNP) shifts from −2.7 V to beyond the measurable voltage range, indicating significant p-doping induced by TOS. Inset: Optical image of a graphene strip with patterned four-probe electrodes used for electrical measurements; scale bar: 20 µm

To effectively achieve p-doping of graphene, we need to first fully oxidize monolayer WSe_2_ into TOS using UV-ozone treatment (Supplementary Information II). Following the oxidation process, a noticeable change in the optical contrast of the WSe_2_ flake was observed, as shown in the inset of Fig. 2a, indicating successful conversion of WSe_2_ to TOS. The oxidation process can be precisely characterized using Raman and photoluminescence (PL) spectroscopy (Fig. 2a, b). For WSe_2_ flakes comprising monolayer and bilayer regions, Raman spectra revealed that the characteristic *E*_2g_ and *A*_1g_ peaks completely disappeared after UV-ozone treatment, indicating full oxidation. PL spectroscopy and spatial mapping further supported this finding. The monolayer region initially exhibited strong PL emission, which was completely quenched after oxidation. Conversely, the bilayer region exhibited the opposite behavior, with initially quenched PL becoming enhanced post-oxidation (Fig. 2b inset). These observations confirm the complete oxidation of the top WSe_2_ layer.

Subsequently, we investigated the doping effect of TOS on graphene using Raman spectroscopy and electrical measurements. Raman analysis of pristine graphene and TOS/graphene reveals a significant reduction in the intensity ratio of the 2D peak to the G peak (*I*_2D_*/I*_G_) as shown in Fig. 2c, decreasing from approximately 3 to 1. We also observed a clear blueshift of the 2D peaks (16 cm^−1^) in TOS/graphene relative to the original position in pristine graphene. These results indicate a hole density of ~ 2 × 10^13 ^cm^−2^ and a Fermi level shift of approximately –500 meV^37^. This degenerate p-type doping satisfies the Pauli blocking condition (2|*E*_F_ |~1.0 eV > 0.8 eV for 1550 nm excitation), effectively suppressing interband absorption and enabling the observed low insertion loss. Furthermore, the sheet resistance values for pristine graphene and TOS/graphene HS, as shown in Fig. 2d, reveal a pronounced shift of the charge neutral point (CNP) from approximately −2.7 V to beyond the measurable voltage range presented. This electrical measurement further confirms that the graphene is heavily p-doped through charge transfer from the adjacent TOS layer.

**Fig. 3:**
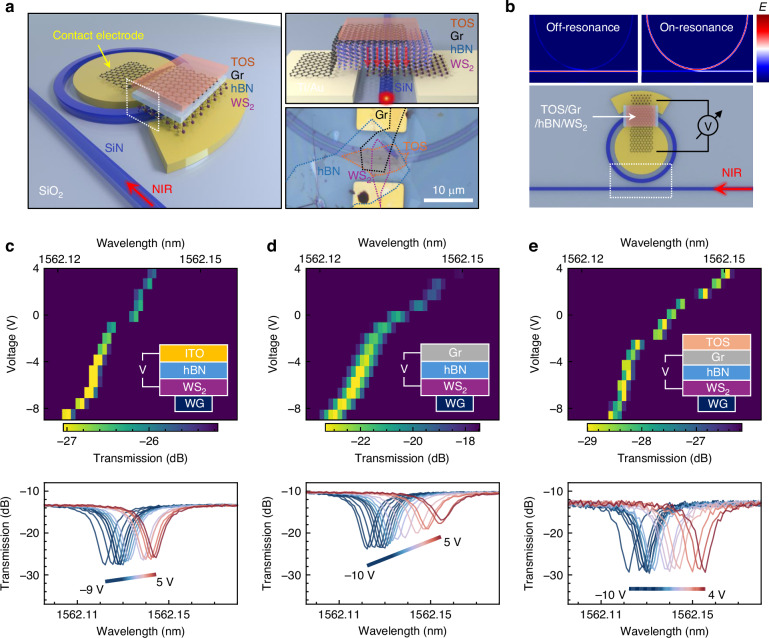
**Phase modulation for device with ITO, graphene, and TOS/graphene electrodes**. **a** Schematic of the fabricated device structure, consisting of a capacitor stack (monolayer WS_2_/hBN/graphene/TOS) integrated onto a SiN microring resonator. Right top: Side-view schematic of the device, highlighting electrical contacts with the bottom WS_2_ layer and top graphene electrode, connected independently to gold electrodes for voltage biasing. Right bottom: Optical image of the actual device. **b** Top: schematic illustrating the on-resonance and off-resonance conditions within the microring-waveguide system. Bottom: Configuration for applying external electrical bias separately to the bottom WS_2_ and top TOS/graphene electrodes. **c**–**e** Top panels: Resonance spectra of microring resonators integrated with **c** ITO/hBN/WS_2_, **d** Gr/hBN/WS_2_, and **e** TOS**/**Gr/hBN/WS_2_ heterostructures, under varying applied bias voltages. Color intensity represents the amplitude of the transmission peaks, with spectral shifts indicating changes in the optical phase. Bottom panels: Corresponding line plots of the transmission spectra extracted from the top panels, highlighting the resonance wavelength shifts and changes in modulation behavior for each electrode configuration

We then investigated the potential of the TOS/graphene HS as a transparent electrode for achieving lossless electro-optic phase modulation. As compared to other TMDs like WSe_2_, monolayer WS_2_ was specifically selected as the active electro-optic material owing to its large bandgap (~2 eV) and efficient tunability of carrier density via external electric fields^30^. The device architecture, as illustrated in Fig. 3a, comprises a capacitor structure with monolayer WS_2_ at the bottom, a 20 nm-thick hBN dielectric spacer, and the TOS/graphene HS serving as the transparent top electrode. The entire device is fabricated on top of a SiN microring, with a 100 nm-thick SiO_2_ cladding layer separating the microring from the HS. This cladding serves to improve the quality of the microring and mitigate strain effects potentially induced by the waveguide (See Materials and methods). Figure 3b outlines the electrical bias configuration, highlighting separate electrode contacts made to the bottom WS_2_ layer and the top TOS/graphene electrode. By applying external bias, we tune the carrier density in WS_2_, thereby inducing changes in its refractive index and subsequently altering the resonance conditions of the microring resonator. Notably, variations in optical absorption of materials on top of the microring resonator can shift the microring resonance from critical coupling toward either under-coupled or over-coupled conditions, resulting in reduced resonance efficiency and modified transmission characteristics (Fig. 3b top).

We systematically compared the electro-optic modulation performance of three different top electrode materials: ITO, graphene, and the TOS/graphene HS. In these three cases, the underlying electro-optic active layer and dielectric insulator were consistently monolayer WS_2_ and hBN, respectively. Figure 3c–e present the experimentally measured transmission spectra of the microring resonators as a function of applied bias voltage for each electrode configuration. The modulation behaviors vary distinctly among these electrode materials (Supplementary Information VII). The ITO electrode exhibits noticeable transmission amplitude variations accompanying its phase modulation, primarily due to inherent optical absorption losses. For graphene electrodes, a substantial phase shift of up to 30 pm is observed. However, this graphene phase modulation causes significant transmission amplitude changes. These changes result from bias-induced tuning of graphene’s Fermi level and corresponding variations in optical absorption. The TOS/graphene electrode achieves not only comparable but enhanced phase modulation performance while simultaneously eliminating detectable amplitude changes. This combination of benefits was previously unattainable using conventional electrode materials^38^.

The extinction ratio, defined as the difference between the maximum and minimum transmission levels, is extracted from the transmission spectra presented in Fig. 3c, d. A comparison across the three devices shows distinct trends. The TOS/graphene electrode exhibits minimal variation in the extinction ratio of 0.08 dB over the entire bias range, indicating negligible bias-induced optical loss. In contrast, the extinction ratio for the graphene-based device decreases significantly with increasing bias (extinction ratio variation = 7.4 dB). reflecting substantial absorption losses. The ITO-based device displays a moderate change in the extinction ratio (2.1 dB), suggesting a bias-dependent but less pronounced optical loss. These observations confirm that TOS/graphene electrodes maintain excellent optical transparency under electro-optical modulation, while conventional graphene and ITO electrodes introduce increased insertion losses due to carrier-induced absorption effects.

**Fig. 4:**
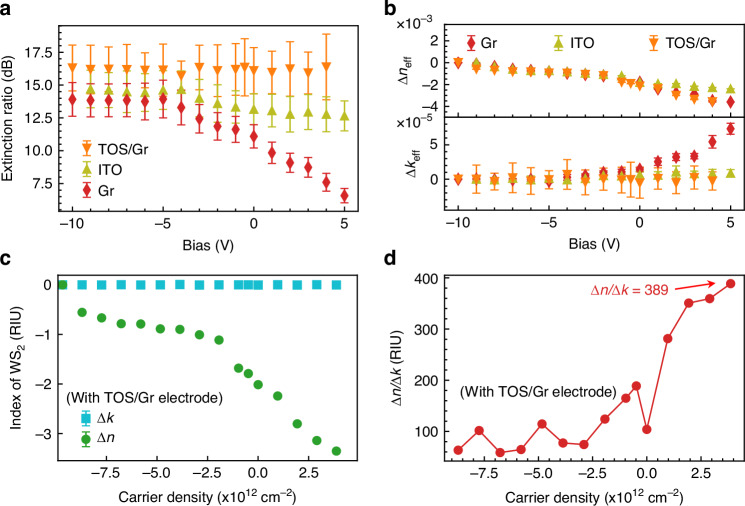
**Electrostatically induced changes in the complex refractive index of monolayer** **WS**_2_ **at NIR wavelengths.** **a** Extinction ratio as a function of applied bias voltage for microring resonators integrated with different top electrodes: TOS/graphene, graphene, and ITO. The extinction ratio is defined as the difference between the maximum transmission and minimum transmission in the spectra, serving as a measure of insertion loss under varying bias conditions. **b** Changes in the real (Δ*n*_eff_) and imaginary (Δ*k*_eff_) components of the effective refractive index of the propagating mode measured in devices employing three different top electrodes: graphene, ITO, and TOS/graphene, as a function of applied voltage. **c** Changes in the intrinsic real (Δ*n*) and imaginary (Δ*k*) parts of the refractive index of monolayer WS_2_ as functions of the electrostatically induced carrier density, obtained by converting Δ*n*_eff_ and Δ*k*_eff_ data from **b**. **d** The ratio of electro-refractive response to electro-absorptive response of monolayer WS_2_ plotted as a function of carrier density (Supplementary Information I), demonstrating the favorable high refractive index modulation relative to absorption changes

We then extract the changes in the real and imaginary components of the effective refractive index of the 2D material-SiN waveguide composite system, denoted as Δ*n*_eff_ and Δ*k*_eff_, respectively, which correspond to carrier-induced refractive index modulation and absorption loss. As shown in Fig. 4b, Δ*n*_eff_ linearly decreases with applied voltage for all three electrode configurations: ITO, graphene, and TOS/graphene, indicating that the carrier density in the active region is modulated in a linear fashion by the external bias. Notably, the graphene and TOS/graphene devices exhibit a larger Δ*n*_eff_ variation over the same voltage range compared to the ITO device. This is attributed to improved interfacial contact and more efficient charge injection at the electrode/WS_2_ interface.

In contrast, the trends observed in Δ*k*_eff_ differ significantly. The Δ*k*_eff_ for the graphene device increases steadily with bias voltage, indicating enhanced optical absorption due to the interaction between the evanescent field of the guided mode and the absorbing graphene layer. Conversely, the ITO and TOS/graphene devices show negligible changes in Δ*k*_eff_ under applied bias, suggesting minimal additional optical absorption. This clear contrast confirms that the graphene electrode directly contributes to insertion loss, whereas the TOS/graphene hybrid electrode remains optically transparent under operation. In the hybrid electrode, the observed phase modulation originates exclusively from the modulated carrier density in the underlying WS_2_.

**Fig. 5:**
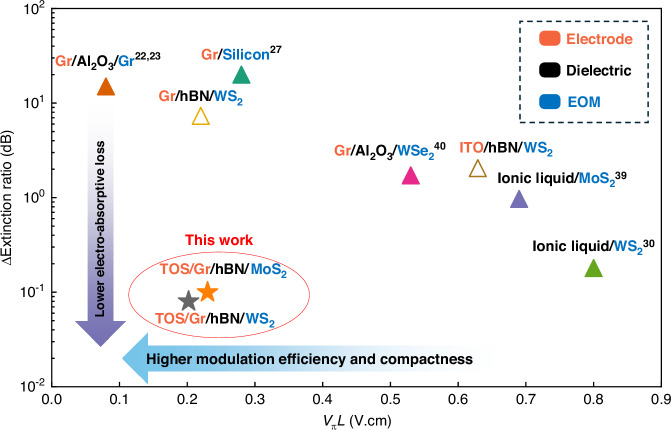
Comparison of extinction ratio variation and half-wave voltage–length product (*V*π·*L*) across various 2D and bulk electro-optic materials. The device with TOS/graphene electrodes in our work exhibits the lowest extinction ratio variation, down to 0.08 dB, surpassing ITO and pristine graphene^22,23^ and ionic liquids^30,39^. This indicates minimal electro-absorptive. Monolayer WS_2_ also shows superior modulation efficiency (*V*π·*L* *=* 0.202 V·cm) compared to silicon^27^, MoS_2_ (Supplementary Information V) and WSe_2_^40^, enabling more compact device designs

The half-wave voltage–length product (*V*π*·L*) was calculated to be 0.629 V·cm, 0.220 V·cm, and 0.202 V·cm for devices with ITO, graphene, and TOS/graphene electrodes, respectively (Supplementary Information VI). These results clearly demonstrate that graphene-based electrodes offer superior capability for modulating the carrier density in WS_2_ compared to conventional ITO electrodes. Furthermore, the modulation efficiency achieved with the TOS/graphene configuration represents an extraordinary advancement that substantially surpasses previously reported values (Fig. 5), establishing a new benchmark for 2D-material-based phase modulators^30,39,40^.

We further extracted the intrinsic complex refractive index changes—Δ*n* and Δ*k*—of the monolayer WS_2_ as a function of electrostatically induced carrier density for the device with TOS/graphene electrodes (Fig. 4c and Supplementary Information IV). Specifically, Δ*n* reaches a maximum modulation of −3.4 RIU at a carrier concentration of 4 × 10^12 ^cm^−2^, while Δ*k* remains negligible across the entire voltage range. This confirms that monolayer WS_2_ exhibits a strong electro-refractive response coupled with minimal electro-absorptive loss at near-infrared wavelengths. The ratio of electro-refractive to electro-absorptive change, *|*Δ*n*|*/|*Δ*k*|, is plotted in Fig. 4d, showing a value of 389, which is significantly higher than previously reported values^30,40^. It is worth noting that this figure of merit could be further enhanced by employing high-κ dielectric layers such as HfO_2_ or Al_2_O_3_^41,42^, which would improve the gate coupling and overall modulation efficiency.

Lastly, we benchmarked the performance of our device against previously reported electro-optic modulators, as summarized in Fig. 5. One key metric is the variation in the extinction ratio—whose values were presented earlier—which reflects electro-absorption losses introduced by the electro-optic active material and electrode layers. Compared to devices using ITO, graphene^22,23^, or ionic liquid gating^30,39^, our TOS/graphene-integrated modulator exhibits the smallest variation in extinction ratio under bias. This indicates minimal optical absorption and highlights its superior optical transparency. The other critical metric (*V*π·*L*) with monolayer WS_2_, demonstrates that it outperforms conventional silicon^27^ and other TMDs such as MoS_2_^39^ and WSe_2_^40^ when used as the active electro-optic material. The combination of the strong electro-refractive response and negligible absorption at telecommunication wavelengths of WS_2_ contributes to superior modulation efficiency and thus enhanced compactness. Together, these advantages establish our platform as a promising candidate for large-scale integration of 2D materials into silicon photonic systems, enabling scalable, low-loss, and high-performance electro-optic modulation for next-generation technologies.

## Discussion

In summary, we have developed a transformative approach using TOS to achieve heavy p-doping of graphene, significantly altering its optical characteristics to achieve near-complete transparency at telecommunication wavelengths. This approach resolves the critical absorption loss problem that has hindered graphene photonic devices for over a decade. Leveraging this TOS/graphene as a transparent top electrode, we developed a compact, near-lossless electro-optic phase modulator that incorporates monolayer WS_2_ as the active modulation layer. The device achieves efficient phase modulation of 0.202 V·cm and exhibits minimal extinction ratio variation of 0.08 dB. These unprecedented results establish not just a promising pathway, but a viable and immediately applicable solution for the seamless integration of 2D materials into silicon photonic platforms in a scalable manner (Supplementary Information VIII). Our demonstration of near-lossless phase modulation enables transformative applications in energy-efficient optical communications, high-speed photonic computing, and robust quantum networks.

## Materials and methods

### SiN waveguide fabrication

A SiN layer of 350 nm was deposited on a SiO_2_ substrate by low-pressure chemical vapor deposition. The SiN waveguide and ring resonator were patterned on AR-P 6200 e-beam resist by electron beam lithography (Raith EPBG 100KV). After developing the resist, the inductively coupled plasma reactive ion etching (ICP-RIE) was used to etch the waveguide and resonator, using CHF_4_ as etching gas and additional Ar to lower the etch rate and maintain the chamber pressure. A plasma asher was used to strip of the remaining resist. The passive sample with SiN waveguide arrays was then cladded with the 1 µm PECVD SiO_2_ and followed by chemical mechanical polishing (CMP). After the CMP process, a 50 nm to 100 nm SiO_2_ cladding with a top surface roughness of <1 nm remained to minimize potentially induced strain effect (Supplementary Information IX). Next, the device was again patterned with positive resist (AZ52143E) and patterned using a direct laser writer (DMO ML 3 Pro) to define the metal electrodes. Ti/Au (10 nm/ 40 nm) was then deposited, followed by liftoff in acetone. Finally, to remove any remaining residue and increase adhesion to our 2D HS, the sample was cleaned using oxygen plasma (FEMTO SCIENCE).

### Transmission spectra measurement

The tunable laser (Santec TSL-510) was used to excite the light in the SiN waveguide (1510–1600 nm). Polarization was controlled using fiber polarization controllers (Thorlabs FPC526) and followed by a tapered waveguide. The light through the tapered waveguide was edge-coupled to the DUT chip waveguide. The 5-axis alignment stage was used to align the input and output ports of the waveguide. The throughput (output) of the waveguide was collected by the tapered waveguide and measured using a Photodetector (High-Dynamic-Range Logarithmic Power Sensors 2103). A source meter (Keithley 2450B) was used for applying the voltage to modulate the transmission using DC probes connected to the electrode of the phase modulator.

### The device fabrication and characterization

TMDs, graphene, and hBN flakes were mechanically exfoliated onto SiO_2_/Si substrates. The thickness of individual flakes was carefully verified via optical contrast using an optical microscope. The HS stack comprising these exfoliated flakes was fabricated using a dry-transfer technique facilitated by a polycarbonate (PC) stamp. Specifically, flakes were first picked up at 120 °C using the PC polymer and subsequently transferred onto the target substrate at 200 °C. After transfer, the residual PC polymer was completely removed by immersing the samples in chloroform for 12 h. For controlled oxidation of as-exfoliated WSe_2_ flakes, UV-ozone treatment was performed at 50 °C for 30 min in the UV ozone cleaner (Samco UV-1), maintaining an oxygen flow rate of 1 L/min, ensuring effective conversion of WSe_2_ into TOS.

PL and Raman spectra were acquired at room temperature using a commercial Raman spectroscopy system (Nanobase XperRam-S567). A 532 nm continuous-wave laser was employed as the excitation source, focused onto the sample through a 100× objective lens. The resulting signals were collected by the same objective lens and analyzed using the built-in spectrometer. The laser power was carefully controlled to avoid sample damage or heating effects during measurements. Spatially resolved PL mapping was performed to characterize the optical quality and homogeneity of the samples before and after oxidation.

The sheet resistance of pristine graphene and TOS-doped graphene samples was characterized using a source-measure unit (SMU, Keithley 2634B). Electrical contacts were fabricated onto the samples using standard lithographic and metal deposition techniques, enabling precise voltage biasing and current measurement. The sheet resistance and corresponding charge neutral point shifts were determined by systematically sweeping the applied gate voltage while measuring the current-voltage characteristics, confirming the doping level and electrical quality of the graphene-based electrodes.

### FDTD simulation

A microring resonator and its adjacent bus waveguide were modeled in Lumerical FDTD by defining precise geometries and assigning accurate material refractive indices, with the simulation domain bounded by perfectly matched layers (PML) to suppress reflections. A broadband optical source was injected into the bus waveguide to excite resonant modes, while strategically placed field monitors recorded the electromagnetic field distributions. A refined mesh was applied, particularly near the resonator, to capture intricate field variations, and the resulting time-domain data was converted to the frequency domain via Fourier transform to extract the transmission spectrum and key resonator parameters such as resonant wavelengths, Q-factor, and free spectral range (FSR).

## Supplementary information

Supporting Information

## Data Availability

The materials, datasets, and code supporting the findings of this study remain unavailable for public access at present but can be obtained by contacting the authors with a suitable request.
