# Supplementary material for: Hybrid tungsten oxyselenide/graphene electrodes for near-lossless 2D semiconductor phase modulators
Source: Light Sci Appl. 2026 Jan 3;15:42. doi: 10.1038/s41377-025-02058-8 (PMC12764534; doi:10.1038/s41377-025-02058-8)
Supplement: Supplementary file 1 — Supporting Information [file 41377_2025_2058_MOESM1_ESM.docx]

**Supplementary Information for**

**Hybrid tungsten oxyselenide/graphene electrodes for near-lossless 2D semiconductor phase modulators**

Shi Guo^1^*, Sung-Gyu Lee^1^*, Xiangxin Gong^1^*, Lalit Singh^1^, Rui Yu^1^, Ahmad Sholehin Bin Juperi^1,2^, Seoungbum Lim^1,2^, Yuhui Yang^1^, Jinpeng Huo^1^, Jeremy Leong^1,2^, Ce Liang^3^, Hyojin Seung^3^, Yangchen He^4^, Daniel Rhodes^4,5^, Min Sup Choi^6^, Takashi Taniguchi^7^, Kenji Watanabe^8^, Wonkeun Chang^1,2^, Tay Beng Kang^1,2^, Luigi Ranno^9^, Juejun Hu^9^, Qingyun Wu^10^, Lay Kee Ang^10^, Jia Xu Brian Sia^1✉^ and Sang Hoon Chae^1,2,11✉^

^1^School of Electrical and Electronic Engineering, Nanyang Technological University, Singapore, 639798, Singapore.

^2^CNRS-International-NTU-Thales Research Alliance (CINTRA), IRL 3288, 50 Nanyang Drive, Singapore, 637553, Singapore.

^3^Pritzker School of Molecular Engineering, University of Chicago, Chicago, IL 60637, USA.

^4^Department of Materials Science and Engineering, University of Wisconsin, Madison, WI, USA.

^5^Department of Physics, University of Wisconsin, Madison, WI, USA.

^6^Department of Materials Science and Engineering, Chungnam National University, Daejeon 34134, Republic of Korea.

^7^Research Center for Materials Nanoarchitectonics, National Institute for Materials Science, Tsukuba, Japan.

^8^Research Center for Electronic and Optical Materials, National Institute for Materials Science, Tsukuba, Japan.

^9^Department of Materials Science and Engineering, Massachusetts Institute of Technology, Cambridge, MA, USA.

^10^Science, Mathematics and technology, Singapore University of technology and design, 8 Somapah Road, Singapore, 487372, Singapore.

^11^School of Materials Science and Engineering, Nanyang Technological University, Singapore, 639798, Singapore.

* These authors contributed equally.

^✉^e-mail: jiaxubrian.sia@ntu.edu.sg, sanghoon.chae@ntu.edu.sg

**Table of Contents**

1. **The thickness of** **hBN dielectric spacer and *I*-*V* curve**
2. **The UV-ozone treatment process for monolayer WSe_2_ and formation of TOS**
3. **DFT simulation for band structure of graphene and TOS**
4. **The model to calculate the** **Δ*n*_eff_ and** **Δ*k*_eff_ for the microring resonator as well as Δ*n*_ws2_ and Δ*k*_ws2_ for monolayer WS_2_ in the TOS/graphene device**
5. **Phase modulation behaviour based on monolayer MoS_2_**
6. ***V*_π*_*L* calculation**
7. **The quality factor for ITO electrode, Gr electrode and TOS/Gr electrode as a function of bias**
8. **Wafer-scale WS_2_ phase modulator fabrication**
9. **Surface of SiN waveguides after Chemical Mechanical Polishing (CMP)**
10. **References**
11. **The thickness of hBN dielectric spacer and *I*-*V* curve**

**
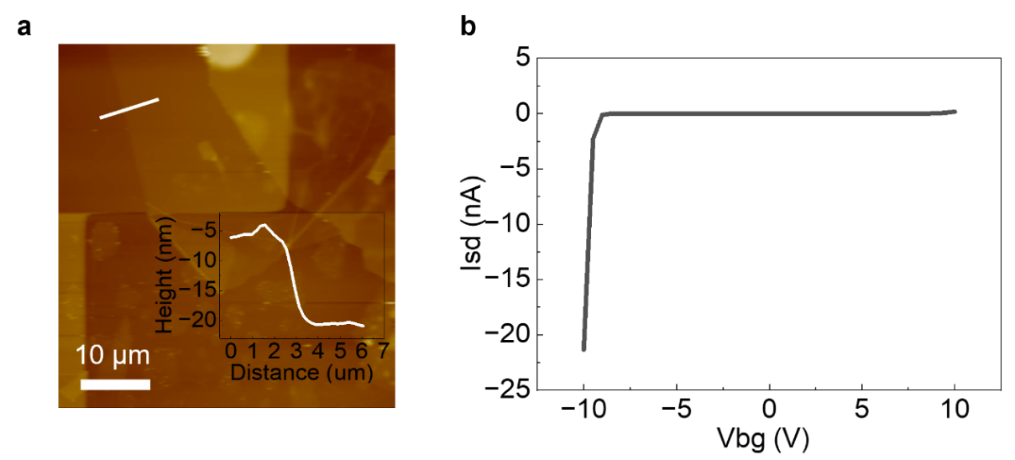
**

**Fig. S1:** **a** Atomic force microscopy (AFM) image of the WS₂/hBN/graphene/TOS heterostructure device, with a white line indicating the position of a height profile measurement across the hBN dielectric layer. Inset: Corresponding height profile revealing the thickness (~20 nm) of the hBN dielectric spacer layer. **b** Current-voltage (I-V) characteristics of the WS₂/hBN/graphene/TOS device, exhibiting typical tunnelling diode behaviour. The observed current asymmetry is attributed primarily to heavy p-type doping of the top graphene electrode by TOS and the inherent structural asymmetry of the device.

We use the equation $n= \epsilon_{0}*\epsilon_{r}*V/qd*{10}^{4}(\mathrm{cm}^{-1})$to convert the applied bias voltage (*V*) to the carrier density *n*, where$\epsilon_{0}$is the vacuum permittivity, $\epsilon_{r}$ is the relative permittivity of the hBN flake, which we take the value of 3.5^1^, *q* denotes the electron charge and *d* is the thickness of hBN flakes, which is determined by AFM in Fig. S1.

1. **The UV-ozone treatment process for monolayer WSe_2_ and formation of TOS**

**_
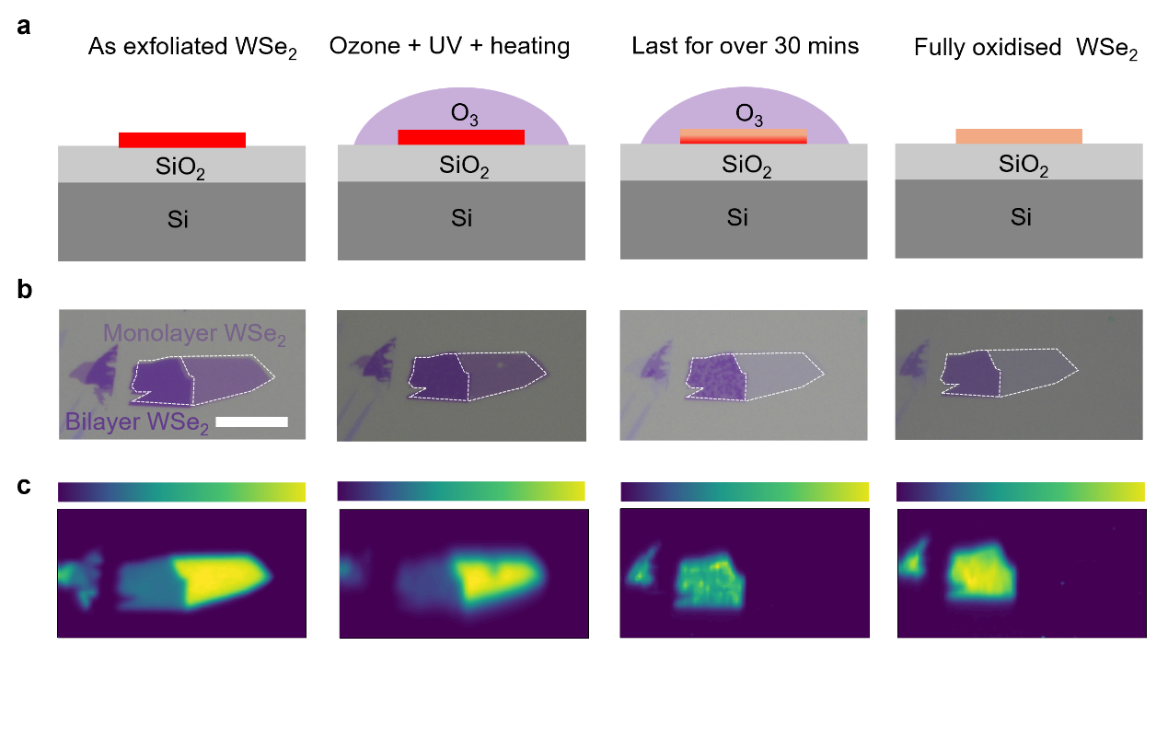
_**

**Fig. S2:** **UV-ozone treatment of WSe_2_ flakes. a** Schematic illustration of the oxidation procedure for WSe_2_ flakes using UV-ozone treatment^2^, clearly depicting the conversion of WSe_2_ into TOS. **b** Optical microscopy images of a WSe_2_ flake composed of monolayer (1L) and bilayer (2L) regions, captured at different stages of the oxidation process; scale bar is 15um. **c** Corresponding photoluminescence (PL) mapping images of the same WSe_2_ flake shown in (b), demonstrating clear evolution of PL intensity as a function of oxidation stage. The significant change in PL intensity confirms the oxidation-induced modification of the electronic and optical properties of the WSe_2_ flake.

**
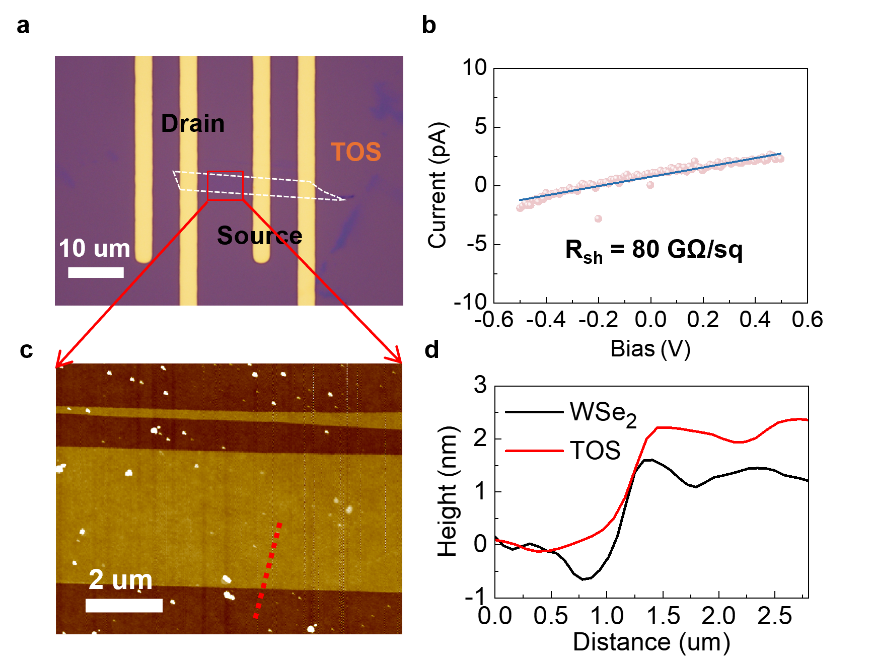
**

**Fig. S3: Sheet resistance and thickness of TOS. a** Optical image of TOS layer with patterned source and drain electrodes. **b** I-V characteristics of the TOS layer, showing an extremely high sheet resistance of approximately 80 GΩ/sq. **c** AFM image of the TOS flake shown in (a), confirming its surface morphology. **d** Thickness measurements of monolayer WSe_2_ before and after UV-ozone treatment along the red dashed line in (c). A slight increase in thickness is observed following the conversion of WSe_2_ to TOS, indicating successful oxidation and formation of the TOS.

1. **Density functional theory (DFT) simulation for band structure of graphene and TOS**

Density functional theory (DFT) simulation was carried out using QuantumATK^3^ to examine the electronic properties of TOS/Gr. A *k*-point mesh of 6 × 12 × 1 was used for the geometry optimization and the property calculations, both based on the Monkhorst–Pack^4^. The atomic force criterion for the geometry optimization was set at 0.01 eV/Å. The generalized gradient approximation (GGA) with the Perdew–Burke–Ernzerhof form (PBE)^5^ was selected for the exchange-correlation functional. The DFT-D3 method with the Grimme scheme was adopted to account for the weak van der Waals interactions in the system. To eliminate interactions from periodic images, a 20 Å thick vacuum layer was inserted between adjacent images.

From the DFT simulation (Fig. S4), the work function difference is about 1 eV, which is consistent with the reported work function value of graphene (4.6 eV) and TOS (5.6 eV).

**
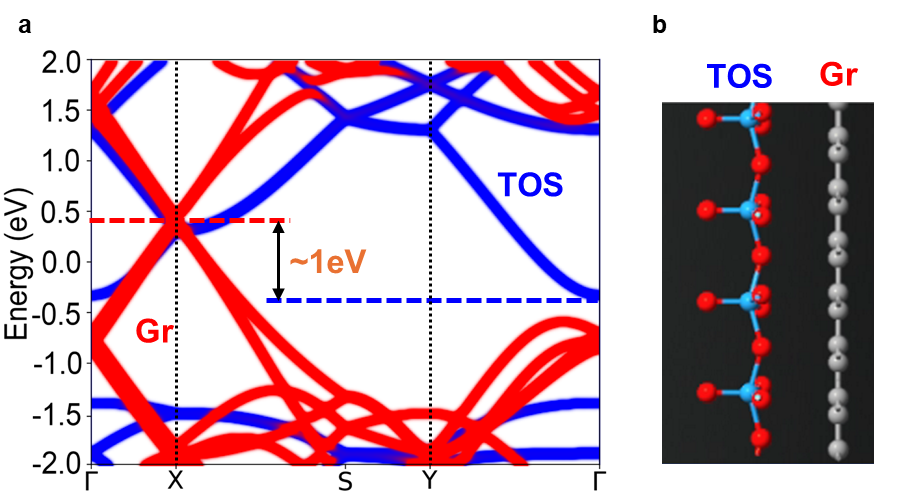
**

**Fig. S4: a** Calculated band structures of graphene (red) and TOS (blue), showing a Fermi level offset of approximately 1 eV, which supports the charge transfer model proposed in this work. **b** The unit cell used in the DFT calculations.

1. **The model to calculate the Δ*n*_eff_ and Δ*k*_eff_ for the microring resonator as well as Δ*n*_ws2_ and Δ*k*_ws2_ for monolayer WS_2_ in the TOS/graphene device**

**4.1. *α* and *t***

**
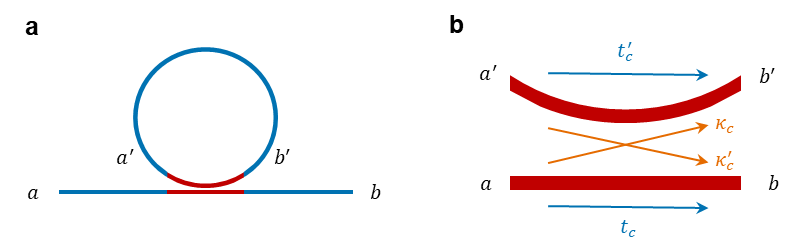
**

**Fig. S5:** **Schematic of a microring resonator.** **a** diagram of the microring resonator. **b** zoomed-in schematic of the directional coupler (DC), showing the denotation for coefficient extraction.

In principle, a microring resonator consists of a DC and a ring waveguide, both of which contribute to optical losses. Fig. S4 illustrates the structure of a typical microring resonator with a directional coupler, along with the associated coupling coefficients. The model used to analyse the resonance condition of the cavity follows the approach described in ref^6,7^. As shown in the zoomed-in view of the directional coupler in Fig. S4b, *t_c_*/*t'_c_* denote the self-coupling coefficients, while *κ_c_*/*κ'_c_* is cross-coupling coefficients. The relationship between the output electric fields *b*/*b'* and the input electric fields *a*/*a'* is expressed as following Eq. (S1) and (S2):

|  | $\boldsymbol{b=}\boldsymbol{t}_{\boldsymbol{c}}\boldsymbol{a+}\boldsymbol{\kappa}_{\boldsymbol{c}}^{\boldsymbol{'}}\boldsymbol{a}^{\boldsymbol{'}}$ | (1) |
| --- | --- | --- |
|  | $\boldsymbol{b}^{\boldsymbol{'}}\boldsymbol{=}\boldsymbol{t}_{\boldsymbol{c}}^{\boldsymbol{'}}\boldsymbol{a}^{\boldsymbol{'}}\boldsymbol{+}\boldsymbol{\kappa}_{\boldsymbol{c}}\boldsymbol{a}$ | (2) |

After the hole round trip, the field *b* is related with *a* by following formula:

|  | $\boldsymbol{a}^{\boldsymbol{'}}\boldsymbol{=}\boldsymbol{t}_{\boldsymbol{r}}^{\boldsymbol{'}}\boldsymbol{b}^{\boldsymbol{'}}$ | (3) |
| --- | --- | --- |

According to Eq. (S1-3), *b* is solved as:

|  | $\boldsymbol{b=a}\left( \frac{\boldsymbol{t}_{\boldsymbol{c}}\boldsymbol{-(}\boldsymbol{t}_{\boldsymbol{c}}\boldsymbol{t}_{\boldsymbol{c}}^{\boldsymbol{'}}\boldsymbol{-}{\boldsymbol{\kappa}_{\boldsymbol{c}}^{\boldsymbol{'}}\boldsymbol{\kappa}}_{\boldsymbol{c}}\boldsymbol{)}\boldsymbol{t}_{\boldsymbol{r}}^{\boldsymbol{'}}}{\boldsymbol{1-}\boldsymbol{t}_{\boldsymbol{c}}^{\boldsymbol{'}}\boldsymbol{t}_{\boldsymbol{r}}^{\boldsymbol{'}}} \right)$ | (4) |
| --- | --- | --- |

Then the factor *t_c_t'_c_ - κ_c_κ'_c_* in Eq. (S4) should be eliminated for the following derivation. Considering the loss introduced by coupler *α_c_* and *α'_c_*, energy conservation was expressed as followings:

|  | $\left\vert\boldsymbol{b} \right\vert^{\boldsymbol{2}}\boldsymbol{+}\left\vert\boldsymbol{b}^{\boldsymbol{'}} \right\vert^{\boldsymbol{2}}\boldsymbol{=}\boldsymbol{\alpha}_{\boldsymbol{c}}^{\boldsymbol{2}}\left\vert\boldsymbol{a} \right\vert^{\boldsymbol{2}}\boldsymbol{+}\boldsymbol{\alpha}_{\boldsymbol{c}}^{\boldsymbol{'2}}\left\vert\boldsymbol{a}^{\boldsymbol{'}} \right\vert^{\boldsymbol{2}}$ | (5) |
| --- | --- | --- |

Using *b* and *b'* from Eq. (S1-2) into Eq. (5) yield Eq. (S6-8):

|  | $\left\vert\boldsymbol{t}_{\boldsymbol{c}} \right\vert^{\boldsymbol{2}}\boldsymbol{+}\left\vert\boldsymbol{\kappa}_{\boldsymbol{c}} \right\vert^{\boldsymbol{2}}\boldsymbol{=}\boldsymbol{\alpha}_{\boldsymbol{c}}^{\boldsymbol{2}}$ | (6) |
| --- | --- | --- |
|  | $\left\vert\boldsymbol{t}_{\boldsymbol{c}}^{\boldsymbol{'}} \right\vert^{\boldsymbol{2}}\boldsymbol{+}\left\vert\boldsymbol{\kappa}_{\boldsymbol{c}}^{\boldsymbol{'}} \right\vert^{\boldsymbol{2}}\boldsymbol{=}\boldsymbol{\alpha}_{\boldsymbol{c}}^{\boldsymbol{'2}}$ | (7) |
|  | $\boldsymbol{t}_{\boldsymbol{c}}^{\boldsymbol{*}}\boldsymbol{\kappa}_{\boldsymbol{c}}^{\boldsymbol{'}}\boldsymbol{+}\boldsymbol{\kappa}_{\boldsymbol{c}}^{\boldsymbol{*}}\boldsymbol{t}_{\boldsymbol{c}}^{\boldsymbol{'}}\boldsymbol{=0}$ | (8) |

Eq. (S7) and Eq. (S8) yield the relation in Eq. (S9). It can be further substituted in Eq. (S4):

|  | $\boldsymbol{t}_{\boldsymbol{c}}\boldsymbol{t}_{\boldsymbol{c}}^{\boldsymbol{'}}\boldsymbol{-}\boldsymbol{\kappa}_{\boldsymbol{c}}\boldsymbol{\kappa}_{\boldsymbol{c}}^{\boldsymbol{'}}\boldsymbol{=}\left( \boldsymbol{t}_{\boldsymbol{c}}^{\boldsymbol{'}}\boldsymbol{t}_{\boldsymbol{c}}^{\boldsymbol{'*}}\boldsymbol{+}\boldsymbol{\kappa}_{\boldsymbol{c}}^{\boldsymbol{'}}\boldsymbol{\kappa}_{\boldsymbol{c}}^{\boldsymbol{'*}} \right)\frac{\boldsymbol{t}_{\boldsymbol{c}}}{\boldsymbol{t}_{\boldsymbol{c}}^{\boldsymbol{'*}}}\boldsymbol{=}\boldsymbol{\alpha}_{\boldsymbol{c}}^{\boldsymbol{'2}}\frac{\boldsymbol{t}_{\boldsymbol{c}}}{\boldsymbol{t}_{\boldsymbol{c}}^{\boldsymbol{'*}}}$ | (9) |
| --- | --- | --- |

the phases *φ_r_* and *φ_c_* are introduced for concise results:

|  | $\boldsymbol{t}_{\boldsymbol{r}}^{\boldsymbol{'}}\boldsymbol{=}\left\vert\boldsymbol{t}_{\boldsymbol{r}}^{\boldsymbol{'}} \right\vert\boldsymbol{e}^{\boldsymbol{i}\boldsymbol{\phi}_{\boldsymbol{r}}^{\boldsymbol{'}}}$ | (10) |
| --- | --- | --- |
|  | $\boldsymbol{t}_{\boldsymbol{c}}^{\boldsymbol{'}}\boldsymbol{=}\left\vert\boldsymbol{t}_{\boldsymbol{c}}^{\boldsymbol{'}} \right\vert\boldsymbol{e}^{\boldsymbol{i}\boldsymbol{\phi}_{\boldsymbol{c}}^{\boldsymbol{'}}}$ | (11) |

And the following coefficients are defined as:

|  | $\boldsymbol{t}\boldsymbol{\equiv}\frac{\left\vert\boldsymbol{t}_{\boldsymbol{c}}^{\boldsymbol{'}} \right\vert}{\boldsymbol{\alpha}_{\boldsymbol{c}}^{\boldsymbol{'}}}$ | (12) |
| --- | --- | --- |
|  | $\boldsymbol{\kappa}\boldsymbol{\equiv}\frac{\left\vert\boldsymbol{\kappa}_{\boldsymbol{c}}^{\boldsymbol{'}} \right\vert}{\boldsymbol{\alpha}_{\boldsymbol{c}}^{\boldsymbol{'}}}$ | (13) |
|  | $\boldsymbol{\alpha}\boldsymbol{\equiv}\left\vert\boldsymbol{t}_{\boldsymbol{r}}^{\boldsymbol{'}} \right\vert\boldsymbol{\alpha}_{\boldsymbol{c}}^{\boldsymbol{'}}$ | (14) |
|  | $\boldsymbol{\phi}\boldsymbol{\equiv}\boldsymbol{\phi}_{\boldsymbol{c}}^{\boldsymbol{'}}\boldsymbol{+}\boldsymbol{\phi}_{\boldsymbol{r}}^{\boldsymbol{'}}$ | (15) |

Then Eq. (S16) can be written as:

|  | $\frac{\boldsymbol{b}}{\boldsymbol{a}}\boldsymbol{=}\left( \frac{\boldsymbol{t-}\boldsymbol{\alpha}\boldsymbol{e}^{\boldsymbol{i}\boldsymbol{\phi}}}{\boldsymbol{1-}\boldsymbol{\alpha}\boldsymbol{t}\boldsymbol{e}^{\boldsymbol{i}\boldsymbol{\phi}}} \right)\frac{\boldsymbol{t}_{\boldsymbol{c}}}{\boldsymbol{t}_{\boldsymbol{c}}^{\boldsymbol{'*}}}\boldsymbol{\alpha}_{\boldsymbol{c}}^{\boldsymbol{'}}\boldsymbol{e}^{\boldsymbol{-i}\boldsymbol{\phi}_{\boldsymbol{c}}^{\boldsymbol{'}}}$ | (16) |
| --- | --- | --- |

Then we can get:

|  | $\boldsymbol{T}\boldsymbol{\equiv}\left\vert\frac{\boldsymbol{b}}{\boldsymbol{a}} \right\vert^{\boldsymbol{2}}\boldsymbol{=}\left\vert\frac{\boldsymbol{t}_{\boldsymbol{c}}}{\boldsymbol{t}_{\boldsymbol{c}}^{\boldsymbol{'*}}} \right\vert^{\boldsymbol{2}}\boldsymbol{\alpha}_{\boldsymbol{c}}^{\boldsymbol{'2}}\left( \frac{\boldsymbol{t}^{\boldsymbol{2}}\boldsymbol{+}\boldsymbol{\alpha}^{\boldsymbol{2}}\boldsymbol{-2}\boldsymbol{\alpha}\boldsymbol{t}\cos\boldsymbol{\phi}}{\boldsymbol{1+}\boldsymbol{\alpha}^{\boldsymbol{2}}\boldsymbol{t}^{\boldsymbol{2}}\boldsymbol{-2}\boldsymbol{\alpha}\boldsymbol{t}\cos\boldsymbol{\phi}} \right)\boldsymbol{=}\left\vert\frac{\boldsymbol{t}_{\boldsymbol{c}}}{\boldsymbol{t}_{\boldsymbol{c}}^{\boldsymbol{'*}}} \right\vert^{\boldsymbol{2}}\boldsymbol{\alpha}_{\boldsymbol{c}}^{\boldsymbol{'2}}\mathcal{T}$ | (17) |
| --- | --- | --- |

Here, the factor $\mathcal{T}$ is represented as Eq. (S18):

|  | $\mathcal{T}\boldsymbol{\equiv}\frac{\boldsymbol{t}^{\boldsymbol{2}}\boldsymbol{+}\boldsymbol{\alpha}^{\boldsymbol{2}}\boldsymbol{-2}\boldsymbol{\alpha}\boldsymbol{t}\mathbf{co}\mathbf{s} \boldsymbol{\phi}}{\boldsymbol{1+}\boldsymbol{\alpha}^{\boldsymbol{2}}\boldsymbol{t}^{\boldsymbol{2}}\boldsymbol{-2}\boldsymbol{\alpha}\boldsymbol{t}\mathbf{co}\mathbf{s} \boldsymbol{\phi}}$ |  | (18) |
| --- | --- | --- | --- |

The transmission spectrum exhibits distinct resonance features as a function of wavelength, characterized by dips at specific resonance wavelengths. At these wavelengths, the optical field is constructively enhanced within the ring resonator. The resonance depth and linewidth are primarily governed by the round-trip loss coefficient α and the self-coupling coefficient 𝑡. The finesse $\mathcal{F}$ is expressed in terms of $\Delta\lambda_{\text{FSR}}$ and $\Delta\lambda_{\text{FWHM}}$ as follows:

|  | $\mathcal{F}\boldsymbol{\equiv}\boldsymbol{\Delta}\boldsymbol{\lambda}_{\text{FSR}}\boldsymbol{/}\boldsymbol{\Delta}\boldsymbol{\lambda}_{\text{FWHM}}$ | (19) |
| --- | --- | --- |

And the extinction ratio is expressed by (Note, the transmission expressed in here is in percentage, not in decibels (dB)):

|  | $\mathcal{E}\boldsymbol{\equiv}\boldsymbol{T}_{\text{max}}\boldsymbol{/}\boldsymbol{T}_{\text{min}}$ | (20) |
| --- | --- | --- |

Then from Eq. (S18), we can get:

|  | $\mathcal{E=}\left[ \frac{\left( \boldsymbol{\alpha}\boldsymbol{+t} \right)\left( \boldsymbol{1-}\boldsymbol{\alpha}\boldsymbol{t} \right)}{\left( \boldsymbol{\alpha}\boldsymbol{-t} \right)\left( \boldsymbol{1+}\boldsymbol{\alpha}\boldsymbol{t} \right)} \right]^{\boldsymbol{2}}$ | (21) |
| --- | --- | --- |
|  | $\cos\left( \frac{\boldsymbol{\pi}}{\mathcal{F}} \right)\boldsymbol{=}\frac{\boldsymbol{2}\boldsymbol{\alpha}\boldsymbol{t}}{\boldsymbol{1+}\boldsymbol{\alpha}^{\boldsymbol{2}}\boldsymbol{t}^{\boldsymbol{2}}}$ | (22) |

By solving the equations, α and *t* can be extracted:

|  | $\left( \boldsymbol{\alpha}\boldsymbol{,t} \right)\boldsymbol{=}\left( \frac{\boldsymbol{A}}{\boldsymbol{B}} \right)^{\boldsymbol{1}\boldsymbol{/}\boldsymbol{2}}\boldsymbol{\pm}\left( \frac{\boldsymbol{A}}{\boldsymbol{B}}\boldsymbol{-A} \right)^{\boldsymbol{1}\boldsymbol{/}\boldsymbol{2}}$ | (23) |
| --- | --- | --- |

With A and B expressed as followings:

|  | $\boldsymbol{A}\boldsymbol{\equiv}\frac{\cos\left( \frac{\boldsymbol{\pi}}{\mathcal{F}} \right)}{\boldsymbol{1+}\sin\left( \frac{\boldsymbol{\pi}}{\mathcal{F}} \right)}$ | (24) |
| --- | --- | --- |
|  | $\boldsymbol{B}\boldsymbol{\equiv}\boldsymbol{1-}\left[ \frac{\boldsymbol{1-}\cos\left( \frac{\boldsymbol{\pi}}{\mathcal{F}} \right)}{\boldsymbol{1+}\cos\left( \frac{\boldsymbol{\pi}}{\mathcal{F}} \right)} \right]\frac{\boldsymbol{1}}{\mathcal{E}}$ | (25) |

Every resonance mode can result in an *α* and *t*. Owning to the DC nature of coupling region, *t* was fitted with sine function, while *α* was fitted with linear function. The cross point of two curve indicates the critical coupling wavelength.

**4.2.** $\boldsymbol{\Delta}\boldsymbol{k}_{\mathbf{eff}_{\mathbf{WS}_{\mathbf{2}}}}$ **and** $\boldsymbol{\Delta}\boldsymbol{n}_{\mathbf{eff}_{\mathbf{WS}_{\mathbf{2}}}}$

According to the propagation of light along waveguide in our proposed device, *α* was defined as $\alpha\left( V \right)=e^{-\frac{2\pi}{\lambda}k_{\mathrm{eff}_{\mathrm{SiN}}}\left( L-L_{\mathrm{WS}_{2}} \right)-\frac{2\pi}{\lambda}k_{\mathrm{eff}_{WS_{2}}}\left( V \right)L_{\mathrm{WS}_{2}}}$. Compared with the state of 0 V where $\alpha\left( 0 \right)=e^{-\frac{2\pi}{\lambda}k_{\mathrm{eff}_{\mathrm{SiN}}}\left( L-L_{\mathrm{WS}_{2}} \right)-\frac{2\pi}{\lambda}k_{\mathrm{eff}_{\mathrm{WS}_{2}}}\left( -1 \right)L_{\mathrm{WS}_{2}}}$, $\Delta k_{\mathrm{eff}_{\mathrm{WS}_{2}}}\left( V \right)$ can be obtained by dividing $\alpha\left( V \right)$ by $\alpha\left( 0 \right)$ as following equation:

|  | $\boldsymbol{\Delta}\boldsymbol{k}_{\mathbf{eff}_{\mathbf{WS}_{\mathbf{2}}}}\left( \boldsymbol{V} \right)\boldsymbol{=}\boldsymbol{k}_{\mathbf{eff}_{\mathbf{WS}_{\mathbf{2}}}}\left( \boldsymbol{V} \right)\boldsymbol{-}\boldsymbol{k}_{\mathbf{eff}_{\mathbf{W}\mathbf{S}_{\mathbf{2}}}}\left( \boldsymbol{V=0} \right)\boldsymbol{=-ln}\frac{\boldsymbol{\alpha}\left( \boldsymbol{V} \right)}{\boldsymbol{\alpha}\left( \boldsymbol{0} \right)}\boldsymbol{*}\frac{\boldsymbol{\lambda}}{\boldsymbol{2}\boldsymbol{\pi}\boldsymbol{L}_{\mathbf{W}\mathbf{S}_{\mathbf{2}}}}$ | (26) |
| --- | --- | --- |

To extract $\Delta n_{WS2}$, we must understand the resonance condition of microring:

|  | $\mathbf{2}\boldsymbol{m\pi=}\frac{\boldsymbol{2}\boldsymbol{\pi}}{\boldsymbol{\lambda}}\boldsymbol{n}_{\mathbf{eff}_{\mathbf{SiN}}}\left( \boldsymbol{L-}\boldsymbol{L}_{\mathbf{W}\mathbf{S}_{\mathbf{2}}} \right)\boldsymbol{+}\frac{\boldsymbol{2}\boldsymbol{\pi}}{\boldsymbol{\lambda}}\boldsymbol{n}_{\mathbf{eff}_{\mathbf{WS}_{\mathbf{2}}}}\left( \boldsymbol{V} \right)\boldsymbol{L}_{\mathbf{W}\boldsymbol{S}_{\boldsymbol{2}}}$ | (27) |
| --- | --- | --- |

By monitoring how the resonance wavelength shifts in response to voltage changes, $\Delta n_{{eff}_{WS2}}$ reads:

|  | $\boldsymbol{\Delta}\boldsymbol{n}_{\mathbf{eff}_{\mathbf{WS}_{\mathbf{2}}}}\left( \boldsymbol{V} \right)\boldsymbol{=}\boldsymbol{n}_{\mathbf{eff}_{\mathbf{WS}_{\mathbf{2}}}}\left( \boldsymbol{V} \right)\boldsymbol{-}\boldsymbol{n}_{\mathbf{eff}_{\mathbf{WS}_{\mathbf{2}}}}\left( \boldsymbol{0} \right)\boldsymbol{=}\frac{\boldsymbol{m}\left( \boldsymbol{\lambda}_{\text{res}}\left( \boldsymbol{V} \right)\boldsymbol{-}\boldsymbol{\lambda}_{\text{res}}\left( \boldsymbol{V=0} \right) \right)}{\boldsymbol{L}_{\mathbf{WS}_{\mathbf{2}}}}$ | (28) |
| --- | --- | --- |

**4.3.** $\boldsymbol{\Delta}\boldsymbol{k}_{\mathbf{WS2}}$ **and** $\boldsymbol{\Delta}\boldsymbol{n}_{\mathbf{WS2}}$

To further characterize the evolution of $\Delta n_{\mathrm{WS}_{2}}$ and $\Delta k_{\mathrm{WS}_{2}}$, FDE simulation was conducted, with WS_2_ flake considered as the dielectric on the top of waveguide. Both $\Delta n_{\mathrm{eff}_{\mathrm{WS}_{2}}}$ and $\Delta k_{\mathrm{eff}_{\mathrm{WS}_{2}}}$ exhibit the roughly linear relationship with $\Delta n_{WS2}$ and $\Delta k_{WS2}$, respectively. The slope is around 0.00107 and 0.001174 as shown in Fig. S5. We used this linear relationship to extract $\Delta n_{\mathrm{WS}_{2}}$ and $\Delta k_{\mathrm{WS}_{2}}$.

**
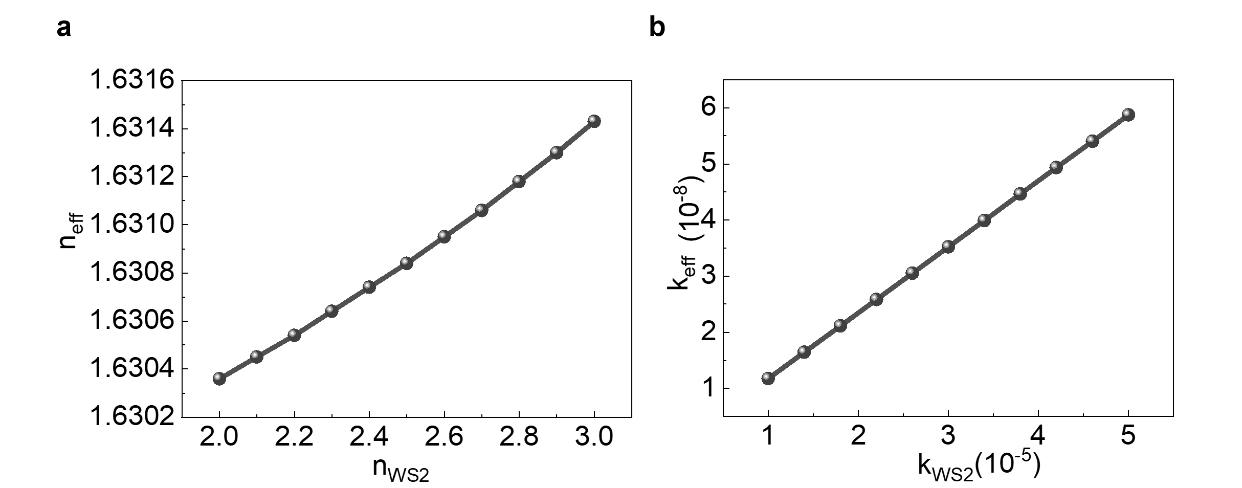
 Fig. S6: The relationship between** $\boldsymbol{\Delta}\boldsymbol{n}_{\mathbf{eff}}$ **and** $\boldsymbol{\Delta}\boldsymbol{k}_{\mathbf{eff}}$ **to** $\boldsymbol{\Delta}\boldsymbol{n}_{\mathbf{W}\mathbf{S}_{\mathbf{2}}}$ **and** $\boldsymbol{\Delta}\boldsymbol{k}_{\mathbf{W}\mathbf{S}_{\mathbf{2}}}$ **respectively.**

1. **Phase modulation behaviour based on monolayer MoS_2_**

As shown in Fig. S7, we have fabricated an additional device with the structure TOS/graphene/hBN/MoS_2_/SiN. The measured result shows a modulation efficiency (*V*π·*L*) of 0.23 V·cm and the extinction ratio variation of 0.1 dB, which are comparable to WS_2_, thereby confirming the reproducibility and generality of our approach.

**
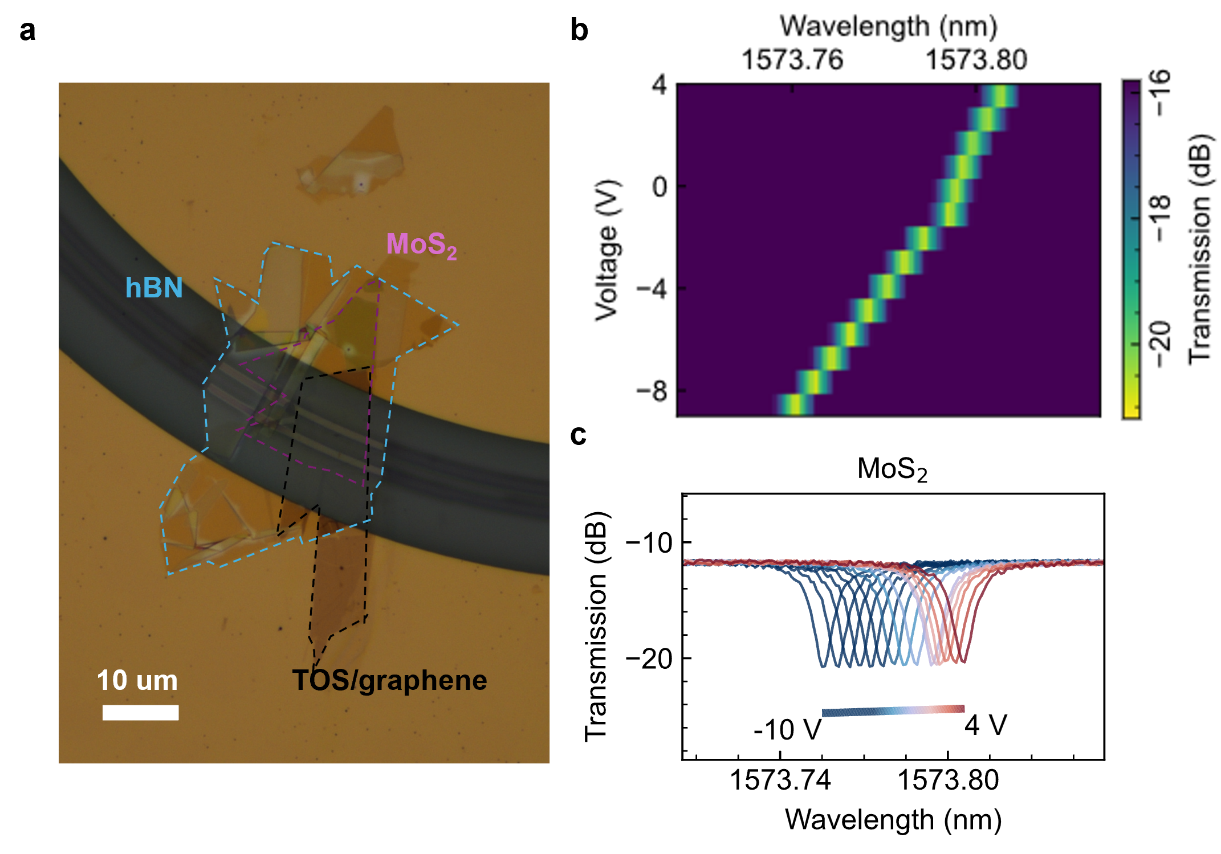
**

**Fig. S7: Phase modulation of the microring resonator with the structure TOS/graphene/hBN/MoS_2_/SiN. a** The optical image of the device. **b** Resonance spectra of microring resonators integrated TOS/Gr/hBN/MoS_2_ heterostructures, under varying applied bias voltages. Colour intensity represents the amplitude of the transmission peaks, with spectral shifts indicating changes in the optical phase. **c** Corresponding line plots of the transmission spectra extracted from (b), highlighting the resonance wavelength shifts and changes in amplitude.

1. ***V*_π_*L* calculation**

The phase shift of light occurred in composite waveguide is expressed as followings:

| $\phi=\frac{2\pi}{\lambda}\cdot n_{eff,\mathrm{WS}_{2}}\cdot L$ | (29) |
| --- | --- |

Because the phase shift only occurs in the active composite waveguide area, the change in phase shift is expressed as following formula:

| $\Delta\phi=\frac{2\pi}{\lambda}\cdot\Delta n_{eff,\mathrm{WS}_{2}}\cdot L$ | (30) |
| --- | --- |

*V_π_* means the voltage required to get the phase shift of π. The phase shift was caused by the effective index change under applied voltage, so we need to evaluate the corresponding effective index change for the phase shift of π as following:

| $\pi=\frac{2\pi}{\lambda}\cdot\Delta n_{eff,\mathrm{WS}_{2},\pi}\cdot L$ | (31) |
| --- | --- |

By fitting the relationship between Δ*n*_eff_ and the applied bias voltage *V* and extracting the corresponding Δ*n*_neff,WS2,π_, the voltage required for a π phase shift (*V*_π_) is determined to be 288 V.

1. **The quality factor for ITO electrode, Gr electrode and TOS/Gr electrode as a function of bias**


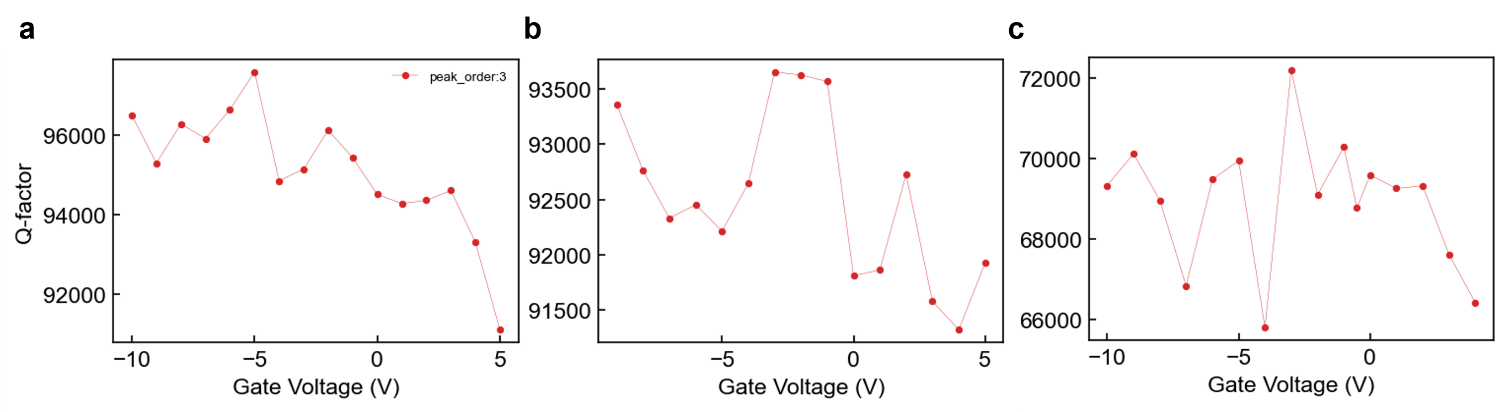


**Fig. S8: Quality factor of the microring resonator as a function of applied bias voltage for devices incorporating graphene (a), ITO (b) and TOS/graphene (c) as electrodes.** **a** Device with graphene electrodes exhibits a clear linear decrease in quality factor with increasing bias, indicating increased optical loss due to bias-induced absorption. **b** ITO-based device also shows a moderate decline in quality factor under bias. **c** the device using TOS/graphene electrodes maintains a nearly constant quality factor despite some fluctuations, confirming the minimal optical absorption and excellent electro-optic transparency of the TOS/graphene electrode.

1. **Wafer-scale WS_2_ phase modulator fabrication**


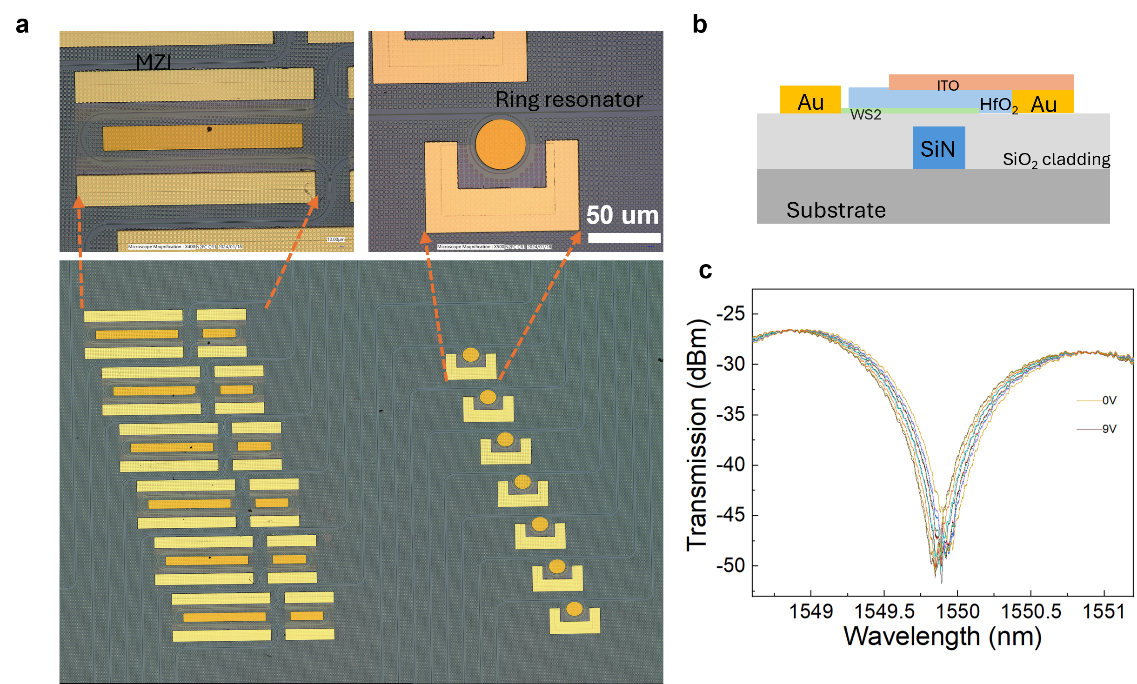


**Fig. S9:** **Wafer scale transfer of MOCVD grown monolayer WS_2_ for the optical phase modulator**. **a** Fabricated SiN waveguide-based arrays of Mach–Zehnder interferometer (MZI) and microring resonators. The microring and the arm of the MZI are covered with large area wet transfer of MOCVD grown WS_2_ and patterned in desired areas. Later a layer HfO_2_ is deposited using atomic layer deposition (ALD) and ITO is deposited using the sputtering. **b** The schematic diagram shows the side view of the active modulator area. The two contacts are made across WS_2_ (monolayer) and ITO (40 nm) separated by the HfO_2_ 20 nm. **c** Transmission spectra from the MZI based modulator showing the change in the intensity on the application of external applied voltage (0 V to 9 V).

1. **Surface of SiN waveguides after Chemical Mechanical Polishing (CMP)**

**
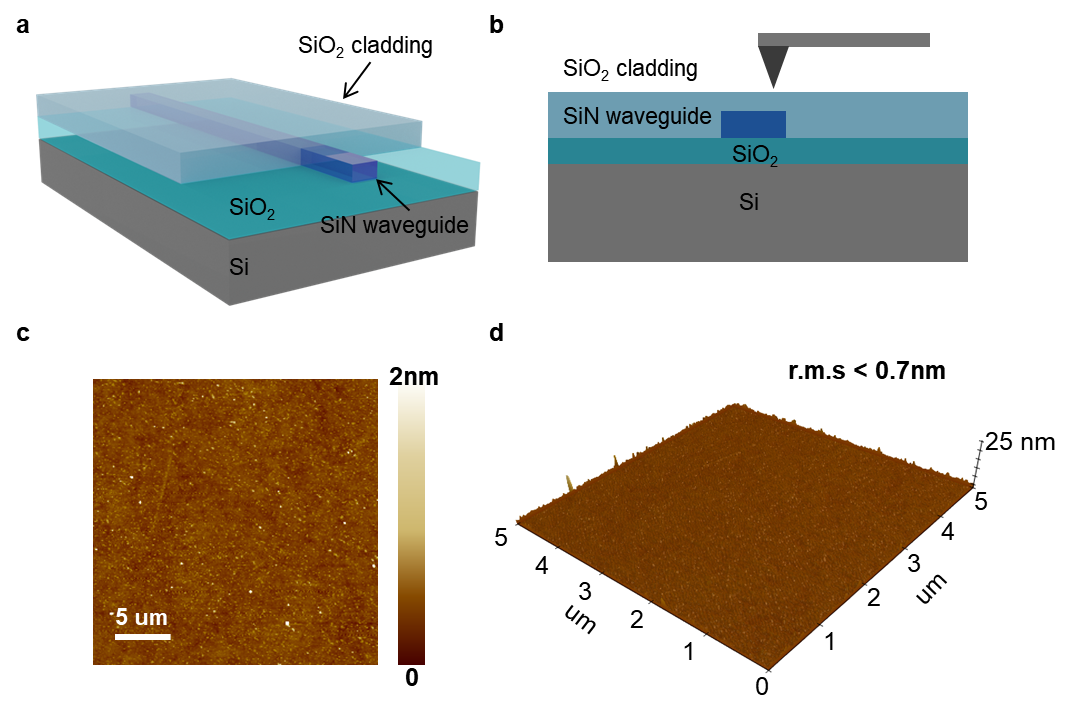
Fig. S10: a** Schematic of the SiN microring resonator (MRR) fabricated on the Si/SiO_2_ substrate with SiO_2_ cladding on top. **b** Side view of MRR and we use AFM to measure the roughness of the surface. **c** AFM image of the surface of a SiO_2_ cladding. **d** 3D view of the surface, showing a surface roughness of less than 0.7 nm.

1. **References**

1 Kim, K. K. *et al.* Synthesis and Characterization of Hexagonal Boron Nitride Film as a Dielectric Layer for Graphene Devices. *ACS Nano* **6**, 8583–8590, doi: 10.1021/nn301675f (2012).

2 Kim, B. S. Y. *et al.* Ambipolar charge-transfer graphene plasmonic cavities. *Nat Mater* **22**, 838-843, doi: 10.1038/s41563-023-01520-5 (2023).

3 Smidstrup, S. *et al.* QuantumATK: an integrated platform of electronic and atomic-scale modelling tools. *Journal of Physics: Condensed Matter* **32**, doi: 10.1088/1361-648X/ab4007 (2019).

4 Monkhorst, H. J. & Pack, J. D. Special points for Brillouin-zone integrations. *Physical Review B* **13**, 5188-5192, doi: 10.1103/PhysRevB.13.5188 (1976).

5 Perdew, J. P., Burke, K. & Ernzerhof, M. Generalized Gradient Approximation Made Simple. *Physical Review Letters* **77**, doi: 10.1103/PhysRevLett.77.3865 (1996).

6 McKinnon, W. *et al.* Extracting coupling and loss coefficients from a ring resonator. *Optics express* **17**, 18971-18982, doi: 10.1364/OE.17.018971 (2009).

7 Yariv, A. Universal relations for coupling of optical power between microresonators and dielectric waveguides. *Electronics Letters* **36**, 321-322, doi: 10.1049/el:20000340 (2000).
